# Supplementary material for: Engineering 3D-Printed Bioresorbable Scaffold to Improve Non-Vascularized Fat Grafting: A Proof-of-Concept Study
Source: Biomedicines. 2023 Dec 18;11(12):3337. doi: 10.3390/biomedicines11123337 (PMC10741522; doi:10.3390/biomedicines11123337)
Supplement: Supplementary file 1 [file biomedicines-11-03337-s001.zip › biomedicines-2705552-supplementary.pptx]

## Slide 1
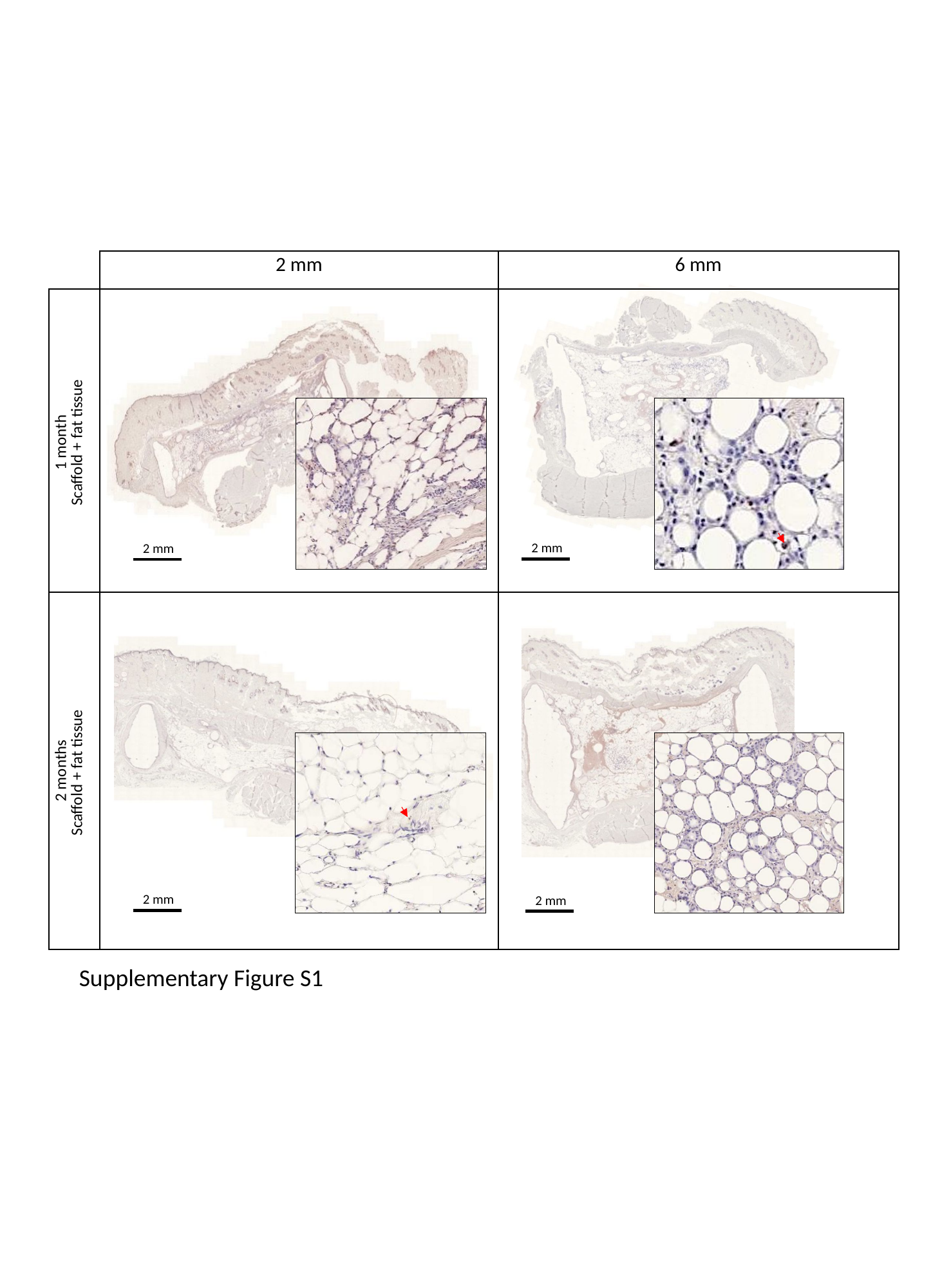

| | 2 mm | 6 mm |
| --- | --- | --- |
| 1 month Scaffold + fat tissue | | |
| 2 months Scaffold + fat tissue | | |
2 mm
2 mm
2 mm
2 mm
Supplementary Figure S1
